# Clinical Efficacy and Safety of the Ketogenic Diet in Patients with Genetic Confirmation of Drug-Resistant Epilepsy

**DOI:** 10.3390/nu17060979

**Published:** 2025-03-11

**Authors:** Ji-Hoon Na, Hyunjoo Lee, Young-Mock Lee

**Affiliations:** Departments of Pediatrics, Gangnam Severance Hospital, Yonsei University College of Medicine, Seoul 06229, Republic of Korea; jhnamd83@yuhs.ac (J.-H.N.); genejoo@yuhs.ac (H.L.)

**Keywords:** ketogenic diet, diet therapy, drug-resistant epilepsy, genetic epilepsies, precision medicine

## Abstract

Drug-resistant epilepsy (DRE) affects 20–30% of patients with epilepsy who fail to achieve seizure control with antiseizure medications, posing a significant therapeutic challenge. In this narrative review, we examine the clinical efficacy and safety of the classic ketogenic diet (cKD) and its variants, including the modified Atkins diet (MAD), medium-chain triglyceride diet (MCTD), and low glycemic index treatment (LGIT), in patients with genetically confirmed drug-resistant epilepsy. These diets induce a metabolic shift from glucose to ketones, enhance mitochondrial function, modulate neurotransmitter balance, and exert anti-inflammatory effects. However, genetic factors strongly influence the efficacy and safety of the cKD, with absolute indications including glucose transporter type 1 deficiency syndrome (GLUT1DS) and pyruvate dehydrogenase complex deficiency (PDCD). Preferred adjunctive applications of the KD include genetic epilepsies, such as *SCN1A*-related Dravet syndrome, *TSC1/TSC2*-related tuberous sclerosis complex, and *UBE3A*-related Angelman syndrome. However, because of the risk of metabolic decompensation, the cKD is contraindicated in patients with pathogenic variants of pyruvate carboxylase and *SLC22A5*. Recent advancements in precision medicine suggest that genetic and microbiome profiling may refine patient selection and optimize KD-based dietary interventions. Genome-wide association studies and multiomics approaches have identified key metabolic pathways influencing the response to the cKD, and these pave the way for individualized treatment strategies. Future research should integrate genomic, metabolomic, and microbiome data to develop biomarker-driven dietary protocols with improved efficacy and safety. As dietary therapies continue to evolve, a personalized medical approach is essential to maximize their clinical utility for genetic epilepsy and refractory epilepsy syndromes.

## 1. Introduction

Drug-resistant epilepsy (DRE), which is defined as the failure of two or more appropriately chosen and tolerated antiseizure medications (ASMs) to achieve sustained seizure freedom, constitutes a significant challenge in epilepsy management [1,2,3]. This condition accounts for 20–30% of all epilepsy cases and has profound implications for the neurodevelopment, cognitive function, and overall quality of life of patients [1,4,5]. Seizures persist despite pharmacological intervention, and frequently lead to delayed milestones, behavioral impairments, and social isolation in DRE [1,6]. Advanced therapeutic strategies, including epilepsy surgery and dietary therapies, such as the ketogenic diet (KD), are frequently employed to manage this refractory condition [1,2]. Among these, the KD, a high-fat, low-carbohydrate diet that induces a state of ketosis, has demonstrated efficacy in significantly reducing seizure frequency and, in some cases, achieving seizure freedom, particularly in pediatric populations. Given the severe neurodevelopmental consequences of untreated DRE, the KD has emerged as a vital non-pharmacological treatment option that warrants further exploration and optimization in clinical practice [1,6,7].

In the 1920s, the KD originated as a therapeutic intervention for DRE based on earlier observations that fasting could alleviate seizures. Wilder first formalized a diet and designed a high-fat, low-carbohydrate regimen to mimic the metabolic effects of fasting and promote ketosis, which is characterized by elevated levels of ketone bodies, such as β-hydroxybutyrate, acetoacetate, and acetone, that serve as an alternative energy source for the brain during glucose deficiency [8]. The neuroprotective mechanisms of KD include reduced neuronal excitability, enhanced GABAergic activity, and modulation of neuroinflammation [9,10]. Recent studies have consistently confirmed the efficacy of the KD in reducing seizure frequency, particularly in pediatric populations with DRE [8]. Furthermore, the safety profile of the KD is well documented, and its side effects, such as gastrointestinal disturbances and hyperlipidemia, are manageable under appropriate clinical supervision. Despite its efficacy and safety, the precise mechanisms whereby KD exerts its anticonvulsant effects remain an active area of research, and emerging evidence highlights its influence on neuro-immuno-metabolism [9,10]. Notably, increasing evidence suggests that the effectiveness and safety of KD may vary depending on the patient’s genetic profile, and this underscores the need for personalized therapeutic approaches [8,9].

Despite its significant efficacy and safety in treating DRE, the variability in the effectiveness of the KD among patients with different genetic backgrounds remains unpredictable [11,12]. Emerging research suggests that genetic mutations, particularly in ion channels such as *SCN1A*, *KCNQ2*, and *STXBP1*, significantly influence both the therapeutic outcomes and tolerability of the KD in DRE [11,13]. However, there is still a lack of comprehensive research into how the KD and its applications interact with the diverse genetic underpinnings of epilepsy, particularly for rare syndromes or complex genetic profiles [13,14].

This review aimed to critically evaluate the clinical efficacy and safety of the KD from a genetics-informed perspective to address the utility of the KD as a personalized therapeutic option for DRE. By synthesizing the current evidence, we aimed to bridge the existing knowledge gaps and provide a framework for future studies that explore the genetic factors that modulate the effectiveness and safety of KD in epilepsy management. To achieve this, a comprehensive literature search was conducted using PubMed, Scopus, and Web of Science databases. Relevant peer-reviewed studies published in English were selected based on their focus on KD and its applications in genetically confirmed DRE. Search terms included “ketogenic diet”, “modified Atkins diet”, “medium-chain triglyceride diet”, “low glycemic index treatment”, “drug-resistant epilepsy”, “genetic epilepsy”, “precision medicine”, and specific gene names associated with KD responsiveness. Studies were screened for relevance, and additional sources were identified through reference list searches. Both clinical and mechanistic studies were included to provide a balanced perspective on efficacy, contraindications, and safety considerations. This approach ensured a thorough synthesis of current knowledge while identifying areas for future research in the personalized application of KD for epilepsy management.

## 2. Ketogenic Diet and Its Applications

The KD is a high-fat, low-carbohydrate dietary intervention that is primarily used to treat DRE. The KD alters the body’s metabolism by enabling a switch from glucose to fat as the primary energy source, wherein ketone bodies that serve as an alternative fuel for the brain are produced, and this bypasses the traditional glycolytic pathways that are impaired in some epileptic conditions. This shift in energy metabolism not only provides an alternative substrate for neuronal energy production, but also exerts multifaceted neuroprotective and anticonvulsant effects via metabolic, neurotransmitter, and neuroinflammatory pathways [15].

The therapeutic potential of the KD is attributed to its ability to enhance mitochondrial bioenergetics and reduce oxidative stress, both of which play critical roles in epileptogenesis. Increased mitochondrial respiration, particularly within astrocytes and neurons, enhances ATP production and improves neuronal resilience under hyperexcitability [16]. Furthermore, this metabolic adaptation modulates ion channel function, stabilizes the neuronal membrane potential, and prevents abnormal depolarization that is associated with seizure activity. Moreover, ketone bodies inhibit histone deacetylases, and this leads to epigenetic modifications that upregulate neuroprotective genes while suppressing pro-epileptogenic pathways.

Beyond the direct metabolic effects, the KD influences key neurotransmitter systems, particularly the homeostasis between inhibitory and excitatory transmission. The KD enhances γ-aminobutyric acid (GABA) synthesis, while reducing glutamate excitotoxicity by modulating glutamic acid decarboxylase and glutamate dehydrogenase activities. This transition favors neuronal inhibition and reduces the hyperexcitability characteristics in DRE. Furthermore, by upregulating the adenosine A1 receptor and further dampening excitatory neuronal activity, ketone bodies interact with adenosine signaling, which is a known anticonvulsant pathway [15,17]. Neuroinflammation, a crucial contributor to DRE, was significantly attenuated by the KD, which reduced the levels of proinflammatory cytokines, such as interleukin-1β and tumor necrosis factor-α, while increasing the levels of anti-inflammatory mediators, such as interleukin-10 [18,19]. These effects contribute to a more stable neural environment and reduce seizure susceptibility. In addition, the KD has been associated with gut microbiota modulation, and studies have indicated that changes in microbial composition may play a role in the anticonvulsant properties of the KD. Certain bacterial populations, such as *Akkermansia muciniphila* and Parabacteroides, increase with the KD and have been linked to enhanced GABAergic activity and metabolic benefits [20].

Despite its well-documented efficacy, the rigid structure of the KD presents challenges in terms of long-term adherence. Over the years, several modifications of the KD have been developed to improve tolerability and dietary compliance while maintaining therapeutic efficacy. Each variant caters to different patient needs and provides options with varying macronutrient compositions and degrees of dietary flexibility [21]. Each of these dietary protocols demonstrated significant efficacy in reducing seizure frequency, particularly in pediatric patients with DRE. The choice of dietary approach is frequently individualized, considering factors such as the patient’s age, genetic background, and metabolic considerations [22,23,24].

### 2.1. Classic Ketogenic Diet

**Figure 1:**
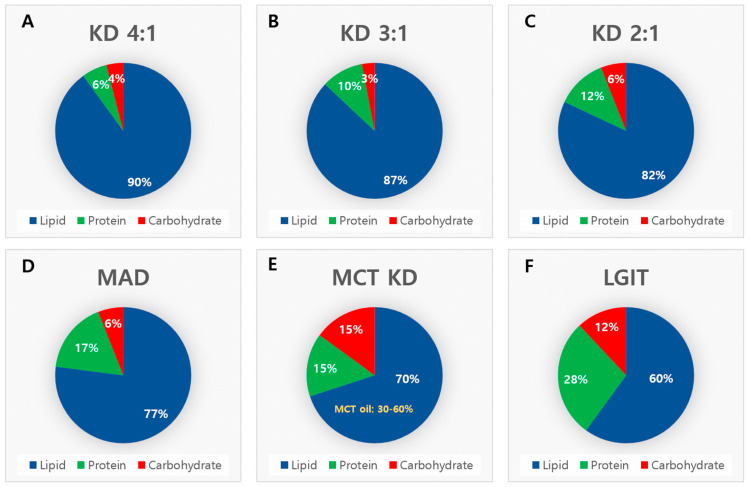
Macronutrient composition of classic ketogenic diet and its applications. KD, ketogenic diet; MAD, modified Atkins diet; MCT, medium-chain triglyceride; LGIT, low-glycemic index treatment. (**A**) KD 4:1, the most stringent form, consists of approximately 90% fat, 6% protein, and 4% carbohydrate, ensuring the highest level of ketosis. (**B**) KD 3:1, a slightly more flexible version, contains 87% fat, 10% protein, and 3% carbohydrate, allowing for increased protein intake while maintaining ketosis. (**C**) KD 2:1, a more lenient adaptation of the classic KD, comprises 82% fat, 12% protein, and 6% carbohydrate, balancing ketosis with improved dietary adherence. (**D**) MAD (Modified Atkins Diet) is a less restrictive alternative, composed of 77% fat, 17% protein, and 6% carbohydrate, making it easier to follow. (**E**) MCT KD (Medium-Chain Triglyceride Ketogenic Diet) includes 70% fat, 15% protein, and 15% carbohydrate, with 30–60% of fat coming from MCT oil, enhancing ketone production with greater dietary flexibility. (**F**) LGIT (Low Glycemic Index Treatment) provides a balanced macronutrient composition of 60% fat, 28% protein, and 12% carbohydrate, focusing on low-glycemic index carbohydrates to stabilize glucose metabolism while offering seizure control benefits.

The Classic Ketogenic Diet (cKD) is the oldest and most rigorously studied form of the ketogenic diet. This diet is traditionally prescribed with a strict 4:1 or 3:1 of fat-to-protein–carbohydrate ratio. The 4:1 version derives approximately 90% of its total energy from fat, 6% from protein, and 4% from carbohydrates, and thereby ensures a high level of ketosis (Figure 1A). A slightly more flexible variation, the 3:1 KD has a reduction in fat content to 87%, with proteins and carbohydrates contributing 10% and 3%, respectively, of the diet (Figure 1B). This adjustment is beneficial for patients who require additional protein intake while maintaining seizure control. Among dietary therapies, the cKD has demonstrated significant success in reducing seizure frequency in children with DRE. Despite its efficacy, the rigid structure of the cKD frequently results in low adherence owing to the limited food options and potential side effects, such as gastrointestinal issues and hyperlipidemia. Careful clinical monitoring is essential to ensure safety and optimize outcomes [23,25,26].

### 2.2. Modified Atkins Diet

As a more flexible version of the classic ketogenic diet (cKD), the 2:1 ketogenic diet consists of approximately 82% fat, 12% protein, and 6% carbohydrate, allowing for a moderate level of ketosis while improving dietary adherence (Figure 1C). Also, the Modified Atkins Diet (MAD) is a practical and less restrictive alternative to the cKD and maintains a 77% fat, 17% protein, and 6% carbohydrate composition, which makes it easier for patients to follow (Figure 1D) [24]. Unlike the cKD, the MAD does not require an initial fasting phase or hospitalization, which makes it suitable for use in older children, adolescents, and adults. The daily carbohydrate intake is limited to 10–20 g in children and up to 25 g in adults, whereas unrestricted protein intake is allowed [25,27].

The MAD has similar efficacy as the cKD in seizure control, with studies reporting seizure freedom in approximately 10% of patients and a significant reduction in seizures in many others [17,22,25]. Moreover, the MAD is associated with fewer adverse effects, such as hypoglycemia and constipation, which improves patient adherence and makes it a viable long-term solution for managing epilepsy [25]. Recent studies have explored the combination of a 2:1 ketogenic ratio within MAD protocols for patients who require greater flexibility while still achieving ketosis. This hybrid approach provides an option for balancing nutritional needs with seizure management [28].

### 2.3. Medium-Chain Triglyceride Diet

A medium-chain triglyceride diet (MCTD) utilizes medium-chain triglycerides (MCTs) derived from sources such as coconut and palm oil. MCTs are more readily absorbed and converted into ketones, which allows for higher carbohydrate and protein intake than the classic KD [2,29,30]. In this version, 70% of the total energy comes from fat, with 15% each from protein and carbohydrates, and 30–60% of fat intake is provided by the MCT oil (Figure 1E). The MCTD improves palatability and compliance, which makes it particularly beneficial for patients who experience gastrointestinal side effects with the cKD. However, careful monitoring is required to manage potential issues such as diarrhea and bloating [25,29,30].

### 2.4. Low Glycemic Index Treatment

The low glycemic index treatment (LGIT) focuses on controlling blood glucose levels by restricting carbohydrate intake to that of foods with a low glycemic index (GI < 50) rather than adherence to a strict ketogenic ratio. The LGIT offers a more balanced macronutrient distribution of 60% fat, 28% protein, and 12% carbohydrates, with a daily carbohydrate allowance of 40–60 g (Figure 1F). LGIT is less restrictive than the other ketogenic variants, which makes it an appealing option for patients who require greater dietary flexibility without compromising seizure control. Although the LGIT may not induce a ketogenic state that is similar to that induced by cKD or MAD, it achieves 50% or greater seizure reduction in many patients [31]. Furthermore, the LGIT’s focus on low-GI foods supports better metabolic control and improves long-term health outcomes [23,25,32].

## 3. Efficacy of the KD and Its Applications in Genetically Derived DRE

The KD and its applications constitute cornerstones in the management of DRE, particularly in patients with DRE induced by genetic causes. Genetic epilepsies, such as developmental and epileptic encephalopathies (DEE), are characterized by early-onset seizures and significant neurodevelopmental impairments that are often resistant to conventional ASMs [33]. Although precision therapies that target specific genetic mutations are still under development, KDs have shown remarkable efficacy against certain genetic forms of DRE. The unique mechanism of KD, which shifts the brain’s energy source from glucose to ketones, bypasses defects in energy metabolism, which are frequently associated with genetic epilepsy. Notably, conditions such as glucose transporter type 1 deficiency syndrome (GLUT1DS), pyruvate dehydrogenase complex deficiency (PDCD), and certain ion channelopathies respond particularly well to the KD by inducing significant seizure reduction and improved neurodevelopmental outcomes. This section explores the efficacy of the KD across various genetic epilepsies, while focusing on the relationship between specific genotypes and therapeutic outcomes [6,8].

### 3.1. Pyruvate Dehydrogenase E1-Alpha Subunit 1

Pyruvate dehydrogenase complex deficiency (PDCD), caused by pyruvate dehydrogenase E1-alpha subunit 1 (*PDHA1*), is the most prevalent form of this mitochondrial disorder and severely affects carbohydrate metabolism. The KD has demonstrated significant efficacy by offering ketone bodies as an alternative energy source that bypasses the defective pyruvate-to-acetyl-CoA conversion [34]. Among the different ketogenic approaches, the MAD, with its 1:1 fat-to-carbohydrate–protein ratio, which is easier to implement than the cKD, offers a more flexible and sustainable option for patients with PDCD while still maintaining therapeutic ketosis [35].

Clinical observations have shown that the MAD is particularly beneficial for patients with milder presentations of PDCD or those who cannot tolerate the strict cKD regimen. Thus, the MAD approach is associated with improved tolerance and fewer gastrointestinal side effects, which makes it a viable alternative to long-term therapies. Furthermore, maintaining plasma beta-hydroxybutyrate levels at 3.0–3.5 mEq/L is crucial to achieve optimal therapeutic outcomes in these patients. Although cKD remains the gold standard, there is increasing evidence that supports the use of MAD in PDCD and highlights the importance of customizing dietary therapy based on individual needs and genetic profiles. Future studies are needed to better understand the long-term efficacy and safety of the MAD compared to other ketogenic strategies for PDCD [36,37].

### 3.2. Solute Carrier Family 2 Member 1

The glucose transporter type 1 deficiency syndrome (Glut1DS) is caused by mutations in the solute carrier family 2 member 1 (*SLC2A1*) gene, which encodes the GLUT1 protein that is responsible for glucose transport across the blood–brain barrier. The resulting energy deficit can lead to seizures, developmental delays, and movement disorders [38]. The KD provides ketone bodies as an alternative energy source that bypasses defective glucose-transport mechanisms. Among Glut1DS patients, the KD is the first-line therapy that significantly improves seizure control, cognitive function, and motor symptoms [39,40].

The MAD offers a more flexible alternative to the classic KD, particularly for adolescents and adults who face challenges in adhering to the strict requirements of the cKD. Although the MAD is less restrictive, it maintains the therapeutic benefits of ketosis by providing sustained seizure reduction. In contrast, the LGIT is not recommended for Glut1DS because of minimal ketone production and lack of proven efficacy under these specific conditions [39]. Long-term adherence to the KD reduces the frequency and severity of seizures, while improving alertness and academic performance, in pediatric patients. Early and consistent dietary interventions are critical to prevent cognitive decline and ensure better developmental outcomes. For patients with *SLC2A1* mutations, the KD remains a cornerstone of therapy that improves seizure outcomes and overall quality of life [41].

### 3.3. Sodium Voltage-Gated Channel Alpha Subunit 1

Pathogenic variants of the sodium voltage-gated channel alpha subunit 1 (*SCN1A*) gene, which encodes the alpha subunit of the voltage-gated sodium channel Nav1.1, lead to impaired inhibitory interneuron function, which results in neuronal hyperexcitability and severe epileptic syndromes, such as Dravet syndrome (DS) [42]. This hyperexcitability is caused by the disruption of sodium channel activity, which reduces GABAergic inhibitory control in the brain, and tips the balance toward excitation. Given the failure of conventional anti-seizure medications to effectively control seizures, the KD has become an essential treatment option for *SCN1A*-related epilepsy [43].

The KD bypasses the dysfunctional sodium channels by providing ketone bodies—β-hydroxybutyrate and acetoacetate—as an alternative energy source for neurons. This shift in metabolism reduces neuronal excitability and stabilizes the cortical networks [44,45]. Furthermore, ketone bodies enhance mitochondrial function and reduce oxidative stress, which is frequently exacerbated by prolonged hyperactivity in *SCN1A*-related disorders. For patients who are unable to adhere to the cKD, the MAD provides a more practical alternative that induces moderate ketosis with less dietary restriction while maintaining significant anticonvulsant effects. Early and sustained dietary interventions for KD or MAD are critical for improving seizure outcomes and preserving cognitive development in patients with *SCN1A* mutations [43,45].

### 3.4. Tuberous Sclerosis Complex 1 and 2

The tuberous sclerosis complex (TSC) constitutes a genetic disorder that is caused by mutations in *TSC1* or *TSC2*, which encode proteins that form the hamartin–tuberin complex that is a key negative regulator of the mTORC1 pathway, which controls cell growth and metabolism [46]. Mutations in these genes lead to chronic mTORC1 activation, which results in cortical malformations, abnormal neuronal growth, and DRE. Compared to *TSC1* mutations, *TSC2* mutations are associated with more severe clinical outcomes, including earlier seizure onset and a greater risk of developmental delays. However, studies have indicated no significant differences in the response to the KD between patients with *TSC1* and *TSC2* mutations [47,48].

By reducing mTORC1 overactivation, improving mitochondrial function, and minimizing oxidative stress, the KD offers a targeted approach to TSC-related epilepsy. Ketone bodies serve as alternative energy substrates that stabilize neuronal membranes and reduce hyperexcitability [49,50]. Clinical evidence has shown that the KD significantly reduces seizure frequency in children with TSC while conferring additional improvements in cognitive and behavioral outcomes. For those who face difficulties in adhering to the cKD, the MAD provides a practical alternative for maintaining moderate ketosis with fewer dietary restrictions. Early initiation of the KD or MAD is essential for optimizing long-term neurodevelopment and preventing neurological deterioration [48,51].

### 3.5. Cyclin-Dependent Kinase-like 5

The cyclin-dependent kinase-like 5 (*CDKL5*) deficiency disorder (CDD) is a rare developmental and epileptic encephalopathy that is caused by mutations in the *CDKL5* gene, which encodes a serine/threonine kinase that is essential for neuronal development, synapse formation, and axonal growth. Mutations in *CDKL5* impair neuronal signaling and lead to early-onset DRE, typically within the first 2 months of life, that is accompanied by severe neurodevelopmental delay [52,53].

By producing ketone bodies that bypass the dysfunctional signaling caused by *CDKL5* mutations, the KD leverages an alternative metabolic pathway that stabilizes neuronal excitability. Clinical data show that the definite response rate to the KD in *CDKL5*-related epilepsy is approximately 18%, with a broader clinical response rate of 50.5%. Although the KD does not correct the underlying genetic mutations, it significantly reduces seizure frequency and improves neurobehavioral outcomes in a substantial proportion of patients. For those who are unable to tolerate the strict cKD regimen, the MAD offers a more flexible yet effective alternative by inducing moderate ketosis while improving adherence and long-term outcomes. Early dietary intervention is critical to optimize seizure control and preserve cognitive function [53,54].

### 3.6. Syntaxin-Binding Protein 1 (STXBP1)

Pathogenic variants of the syntaxin-binding protein 1 (*STXBP1*) disrupt synaptic vesicle release by impairing the SNARE complex, which is essential for neurotransmitter exocytosis. Synaptic dysfunction causes severe developmental delays, early-onset seizures, and variable neurocognitive outcomes in affected individuals. The underlying mechanism, which is of haploinsufficiency, reduces the availability of the functional Munc18-1 protein and leads to impaired inhibitory control and neuronal hyperexcitability. Traditional ASMs provide temporary relief; however, seizures frequently become refractory and necessitate alternative approaches [55]. In this context, the KD is a novel therapeutic strategy. By switching the brain’s primary energy source from glucose to ketones, the KD bypasses dysfunctional synaptic signaling and reduces neuronal excitability. Furthermore, emerging evidence suggests that ketones may directly modulate synaptic activity and enhance mitochondrial function, to thereby improve cellular resilience against hyperexcitability [55,56]. Interestingly, *STXBP1*-related epilepsy appears to respond variably to the KD, and the response depends on the severity of the mutation and the clinical phenotype. Although not universally effective, the KD provides meaningful seizure reduction in a significant subset of patients. This metabolic approach that focuses on cellular stabilization represents an important step in the personalized treatment of *STXBP1* disorders, and shifts the focus from symptom control to addressal of the underlying synaptic dysfunction [56].

### 3.7. Ubiquitin Protein Ligase E3A

Pathogenic variants of the ubiquitin protein ligase E3A (*UBE3A*) cause the Angelman syndrome (AS), which is a neurodevelopmental disorder that is frequently associated with ASM-resistant DRE. AS-related epilepsy is particularly challenging, because individuals with maternal deletions tend to experience more severe drug-resistant seizures [57,58,59]. Recent studies demonstrated that the cKD and its applications, including the LGIT, can significantly improve seizure control in patients with the AS. The efficacy of the cKD in the AS may be attributed to its metabolic and neuroprotective effects. The AS is characterized by an imbalance between excitatory and inhibitory neurotransmission, which is partly attributable to reduced GABAergic signaling. The cKD increases brain GABA levels, restores inhibitory control, and reduces seizure susceptibility [57,60]. A retrospective review of patients with AS treated with LGIT revealed that the majority experienced seizure reduction, with 22% achieving complete seizure freedom and 43% maintaining seizure control, except during illness. Furthermore, case studies of the cKD in the AS-related NCSE have reported resolution of seizures within days of the diet initiation, suggesting a rapid mechanism beyond ketone metabolism [60,61]. The favorable safety profile of dietary therapies makes them suitable for long-term treatment, particularly in patients with severe epilepsy who fail conventional ASM-based treatment [59,60].

### 3.8. Mitochondrial-Encoded tRNALeu (UUR) 1

Pathogenic variants of the *MT-TL1* (Mitochondrially Encoded tRNA^Leu(UUR)^) gene, most notably the m.3243A>G variant, are the primary genetic causes of the mitochondrial encephalopathy, lactic acidosis, and stroke-like episode (MELAS) syndrome. This mutation impairs mitochondrial protein synthesis, particularly that of oxidative phosphorylation (OXPHOS) complexes I and IV, which are crucial for ATP production. The resulting energy deficit triggers a cascade of neurological and metabolic complications, including seizures, stroke-like episodes, and progressive neurodegeneration [62,63]. The KD offers a promising therapeutic strategy by providing ketone bodies as an alternative energy source, bypassing the dysfunctional glycolytic pathway, and restoring mitochondrial function. Ketones enhance the stability and activity of the OXPHOS complex I, reduce the NADH/NAD+ ratio, and improve mitochondrial respiration [62]. In a case study of a patient with MELAS with an m.3243A>G mutation, long-term KD therapy significantly reduced seizure frequency, improved cognitive function, and delayed stroke-like episodes [64]. Moreover, the KD promotes mitochondrial biogenesis and increases the wild type mtDNA copy number, to further stabilize cellular energy metabolism. Although evidence of other KD variants, such as MAD or MCTD, in *MT-TL1*-related disorders is limited, standard KD remains a crucial management option for therapy-refractory epilepsy and cases with MELAS-associated systemic symptoms. Early and sustained KD interventions can dramatically improve clinical outcomes, and this underscores the importance of customized metabolic therapies for mitochondrial diseases [63].

### 3.9. Other Genes

Pathogenic variants of *PCDH19*, *SCN8A*, *KCNQ2*, and *GRIN2A* are associated with distinct forms of developmental and epileptic encephalopathy (DEE), many of which present with DRE. In *PCDH19*-related epilepsy, which primarily affects females, clustered seizures frequently progress to refractory status epilepticus (SE), wherein conventional treatments fail. The KD significantly reduces SE episodes and thus offers a critical alternative therapy for the management of *PCDH19*-related epilepsy [65]. The underlying therapeutic mechanism involves improved neuronal stability and reduced excitatory activity in the cortical networks [66].

In *SCN8A* encephalopathy, pathogenic variants lead to gain-of-function mutations in the *SCN8A* gene, and this results in persistent sodium currents through the NaV1.6 channel and increased neuronal excitability [67]. The KD stabilizes neuronal activity by reducing persistent sodium currents and improving mitochondrial function, and thereby provides seizure control in patients who are unresponsive to sodium channel-blocking medications. Some studies have suggested that *SCN2A*-related DEE may respond to the KD as a treatment for seizure control [68,69,70]. Although some patients with pathogenic variants of *SCN2A* experience seizure reduction with the KD, the evidence remains limited, and further research is needed to clarify the efficacy and long-term benefits of the KD [11]. *KCNQ2*-related epileptic encephalopathy originates from variants that affect potassium channel function, which leads to impaired neuronal repolarization and increased excitability. The ketone body β-hydroxybutyrate, which is produced during treatment with the KD, directly activates *KCNQ2/3* channels and enhances M-current, which stabilizes membrane potential and reduces hyperexcitability. Channel activation plays a pivotal role in controlling seizures in patients with *KCNQ2* [71,72]. Pathogenic variants of the *GRIN2A* are associated with the Landau–Kleffner syndrome (LKS), which is a rare epileptic encephalopathy characterized by language regression and focal epilepsy [73]. Despite potential benefits of the KD in some cases of LKS, there is limited evidence supporting its efficacy in *GRIN2A*-related epilepsy. Given the broad phenotypic spectrum of *GRIN2A* mutations, further research is needed to determine whether the KD and its applications constitute a targeted therapy for these patients [73,74].

Although the underlying mechanisms vary, the cKD offers a metabolic approach that bypasses defective pathways, stabilizes synaptic activity, and protects against neurodegeneration (Table 1). Expanding the use of the KD variants, such as the MAD, MCTD, and LGIT, could further enhance their clinical usefulness in genetically complex conditions. Future research on genotype-specific responses could refine and personalize these interventions to achieve optimal outcomes.

## 4. Safety of Ketogenic Diet and Its Applications

The safety profiles of the KD and its applications, including MAD, MCTD, and LGIT, have been extensively studied, and the results underscore both short-term and long-term considerations [2,23,25,30,31]. Although these dietary therapies provide significant benefits to patients with DRE, they are associated with risks. Early complications of the KD, such as gastrointestinal disturbances (e.g., constipation, nausea, and vomiting), dehydration, and symptomatic hypoglycemia, are common, particularly during the initiation phase [8,17]. The MAD and LGIT generally exhibit fewer early complications owing to their less restrictive nature, which makes them more tolerable for many patients [8,84].

Comparative studies have shown that the risks of long-term complications differ among dietary therapies [2,17]. The cKD is associated with a high incidence of nephrolithiasis, hyperlipidemia, and micronutrient deficiencies, with nephrolithiasis occurring in approximately 7% of pediatric patients. Potassium citrate supplementation significantly reduces the risk of nephrolithiasis; however, the regular monitoring of kidney function is essential [17]. By contrast, the MAD is associated with fewer renal complications and better growth outcomes, which makes it the preferred option for older children and adolescents. Although the MCTD is effective in promoting ketosis, it can lead to gastrointestinal intolerance and diarrhea owing to the high intake of MCTs [2,29]. Bone health is a critical concern, particularly in children who are treated with long-term KD. Reduced bone mineral density in patients after prolonged therapy increases the fracture risk, and routine assessment of calcium and vitamin D levels and bone density is recommended to mitigate this risk. The MAD and LGIT pose a lower risk to bone health because of their balanced macronutrient profiles [17,84]. Beyond these general risks, certain genetic conditions further influence the safety and tolerability of the KD and its variants. Specific pathogenic variants, particularly those that affect mitochondrial function, fatty acid oxidation, and carnitine transport, can predispose individuals to severe metabolic complications, and this necessitates a highly individualized approach to dietary therapy (Table 2) [8,85,86].

Certain patient populations require the cautious implementation of these therapies. Mitochondrial disorders (MDs) have historically been considered contraindications for the KD; however, recent studies have suggested genotype-specific responses in patients with MDs [87]. Patients with mitochondrial DNA (mtDNA) deletions generally experience severe adverse effects following the KD. Studies have documented cases of rhabdomyolysis, worsening myopathy, and metabolic crises, which make the KD unsuitable for this population. The inability of mitochondria to efficiently utilize fatty acids for energy owing to impaired oxidative phosphorylation leads to an increased reliance on anaerobic metabolism, which exacerbates lactic acidosis and energy deficits. In addition, prolonged ketosis in these patients can compromise mitochondrial function and worsen neuromuscular symptoms. However, some reports suggest that the LGIT, which provides greater glucose availability, may constitute a safer alternative for specific patients with mtDNA deletions by minimizing the metabolic stress associated with the cKD [63,78]. In contrast, the pathogenic variant of *POLG* presents a complex scenario, in which some cases report transient seizure improvement under the KD, although long-term outcomes remain poor, with significant risks of metabolic decompensation, liver dysfunction, and even fatal outcomes. Owing to these risks, the KD should be used with extreme caution in patients with pathogenic variants of *POLG*, and its use should be considered only under strict clinical monitoring. Given the metabolic instability associated with these conditions, alternative therapeutic strategies, such as the LGIT or modified dietary approaches, may be preferable to reduce the risk of metabolic crises whereas offering seizure-control benefits [63,83].

Among the nuclear DNA mutations, patients with *PDHA1*-related PDCD benefit from the KD by mechanisms that bypass pyruvate-oxidation defects [63]. Despite risks, patients with therapy-refractory epilepsy may still benefit from the KD when implemented with rigorous monitoring. A recent 2024 study on patients with mitochondrial myopathy demonstrated that the MAD was better tolerated than the classic KD, with fewer severe adverse effects and a longer duration of adherence [78]. This highlights the potential role of KD variants in reducing risk, while maintaining therapeutic benefits. Further research is essential to identify biomarkers that predict KD responsiveness and safety in MD subtypes, to ensure a personalized approach to dietary therapy [63,78,84].

Pathogenic variants of *SLC22A5* cause primary carnitine deficiency (PCD), a metabolic disorder that impairs cellular transport of carnitine and leads to defective fatty acid oxidation [88]. This condition results in severe metabolic consequences, including hypoketotic hypoglycemia, cardiomyopathy, and intractable epilepsy, as observed in documented cases [77]. As fatty acid oxidation is disrupted, the KD, which relies on fat metabolism for energy production, is contraindicated in PCD. Patients with PCD require lifelong carnitine supplementation to prevent metabolic decompensation, and this necessitates alternative dietary approaches for seizure management [8]. Pyruvate carboxylase (PC) deficiency is caused by pathogenic variants in the *PC* gene and is an autosomal recessive metabolic disorder that affects gluconeogenesis, the citric acid cycle, and neurotransmitter synthesis. PC deficiency manifests as severe metabolic acidosis, hyperammonemia, and neurological dysfunction, including epilepsy [75]. Given that PC deficiency results in the impaired anaplerotic replenishment of oxaloacetate, patients rely heavily on glucose metabolism for energy. The KD is contraindicated in these individuals as it shifts metabolism away from glucose utilization, exacerbates metabolic acidosis, and worsens neurological symptoms [75,76].

Pathogenic variants in *ACADVL*, *ACADM*, *ACADS*, *HADHA*, and *HADHB* disrupt key enzymes that are involved in fatty acid β-oxidation, and this leads to metabolic disorders such as very long-chain acyl-CoA dehydrogenase deficiency (VLCADD), medium-chain acyl-CoA dehydrogenase deficiency (MCADD), short-chain acyl-CoA dehydrogenase deficiency (SCADD), and long-chain 3-hydroxyacyl-CoA dehydrogenase deficiency (LCHADD) conditions, wherein the cKD is contraindicated owing to the body’s inability to efficiently metabolize fatty acids, which increases the risk of hypoglycemia, metabolic acidosis, and energy deficiency [80,81,82,89,90]. However, when long-chain fatty acid metabolism is impaired, medium-chain fatty acids can still be partially utilized and MCT supplementation may provide an alternative energy source. Therefore, the MCTD should be carefully considered when selecting patients under close metabolic monitoring, whereas other fatty acid oxidation disorders constitute absolute contraindications for the KD [90].

**Figure 2:**
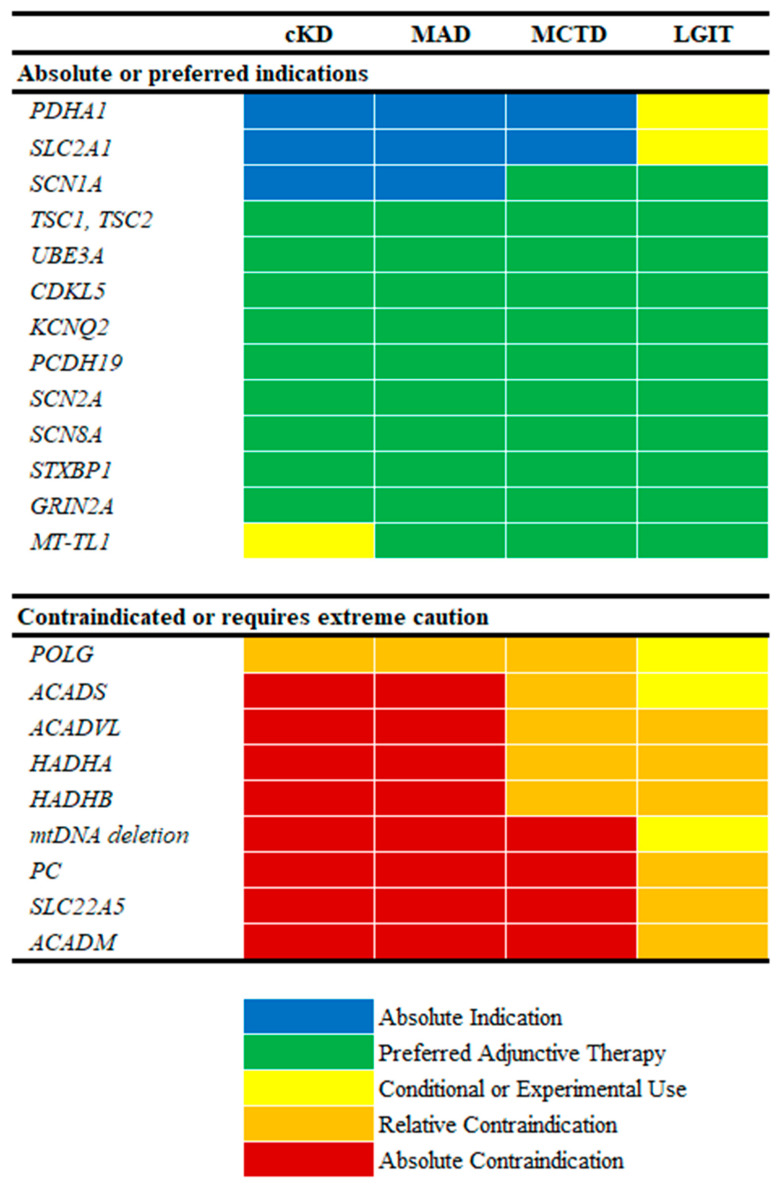
Genotype-specific indications and contraindications for the cKD and Its applications in confirmed drug-resistant epilepsy with genetic causes. cKD, classic ketogenic diet; MAD, modified Atkins diet; MCTD, medium-chain triglyceride diet; LGIT, low glycemic index treatment; PDHA1, pyruvate dehydrogenase E1-alpha subunit 1; SLC2A1, solute carrier family 2 member 1; SCN1A, sodium voltage-gated channel alpha subunit 1; TSC1, tuberous sclerosis complex 1; TSC2, tuberous sclerosis complex 2; UBE3A, ubiquitin protein ligase E3A; CDKL5, cyclin-dependent kinase-like 5; KCNQ2, potassium voltage-gated channel subfamily Q member 2; PCDH19, protocadherin 19; SCN2A, sodium voltage-gated channel alpha subunit 2; SCN8A, sodium voltage-gated channel alpha subunit 8; STXBP1, syntaxin-binding protein 1; GRIN2A, glutamate ionotropic receptor NMDA type subunit 2A; MT-TL1, mitochondrially encoded tRNA leucine 1; POLG, DNA polymerase gamma; ACADS, short-chain acyl-CoA dehydrogenase; ACADVL, very long-chain acyl-CoA dehydrogenase; HADHA, hydroxyacyl-CoA dehydrogenase alpha subunit; HADHB, hydroxyacyl-CoA dehydrogenase beta subunit; mtDNA, mitochondrial DNA; PC, pyruvate carboxylase; SLC22A5, solute carrier family 22 member 5; ACADM, medium-chain acyl-CoA dehydrogenase.

Figure 2 categorizes the genotype-specific indications and contraindications for the cKD and its applications, which highlights the conditions wherein dietary therapy is strongly recommended, considered adjunctive, experimental, relatively contraindicated, or absolutely contraindicated, based on the underlying metabolic and genetic factors. Despite these safety concerns, most families report an overall improvement in quality of life with the KD and its variants, particularly when seizures are well controlled. However, individualized risk assessment is essential. The KD may not be suitable for patients with pre-existing metabolic disorders, compromised liver function, or severe gastrointestinal diseases. In such cases, the MAD or LGIT may offer safer alternatives while preserving therapeutic benefits [17,23]. The success of dietary therapy depends heavily on multidisciplinary care with neurologists, dietitians, and other specialists working closely to monitor and adjust the diet according to the patient’s evolving needs. Routine laboratory monitoring and follow-up are crucial for early identification and management of complications. Future research should focus on refining dietary protocols and exploring the genetic predictors of adverse events to improve patient safety and expand the use of these therapies [8,23,25].

## 5. Limitations and Future Directions

Despite its well-documented efficacy against DRE and other neurological disorders, the KD has several limitations that require further investigation. One of the most critical challenges is the interindividual variability in responses to the KD, which suggests a strong genetic and metabolic influence on the effectiveness of the KD [79]. Some patients may experience limited seizure reduction or significant metabolic side effects, and this indicates that dietary therapies must be customized to individual metabolic and genetic profiles [91]. Furthermore, the long-term metabolic consequences of the KD, particularly in pediatric and older populations, require careful monitoring. Issues such as growth retardation, bone mineral density loss, dyslipidemia, and nephrolithiasis remain concerns in patients undergoing prolonged KD therapy [79,92].

One emerging area of research focuses on the role of β-hydroxybutyrate (BHB), the primary ketone body produced during the KD, in mediating its neuroprotective effects. Recent studies have indicated that BHB is not merely a metabolic fuel but also a signaling molecule that influences gene expression, mitochondrial function, and neurotransmitter balance [92,93]. Mitochondrial bioenergetics is enhanced by BHB, which increases ATP production and improves neuronal resilience under hyperexcitatory conditions [94]. Moreover, BHB acts as a histone deacetylase inhibitor and modulates epigenetic processes that may contribute to its long-term neuroprotective effects. However, the long-term effects of sustained BHB elevation on neuronal function and metabolic homeostasis are not yet fully understood and require further investigation [92,94].

Another major limitation is the lack of precision in predicting responsiveness to KD across different genetic epilepsy syndromes. Although the KD is considered the first-line treatment for *GLUT1* deficiency syndrome, its efficacy in other genetic epilepsies, such as *SCN1A*, *SCN8A*, *KCNQ2*, and *POLG*-related disorders, remains highly variable [91]. In some cases, such as mitochondrial disorders, the KD has historically been considered contraindicated because of concerns about exacerbation of metabolic dysfunction [79]. However, recent studies suggest that patients with certain mitochondrial mutations may still benefit from the KD, particularly those that impair glucose metabolism but retain the ability to utilize ketone bodies [92,93]. This paradigm shift highlights the need for precision medicine approaches that integrate genomic, metabolomic, and transcriptomic analyses to optimize dietary therapies for the individual patient [91].

The integration of the KD into mainstream clinical practice is complicated by adherence and patient tolerability. Many families struggle with the rigid dietary restrictions associated with the KD, and this leads to suboptimal compliance and treatment discontinuation [79]. To address this, researchers are exploring alternative ketogenic strategies, such as MAD, MCTD, and LGIT, which offer greater flexibility while still inducing ketosis [91]. Furthermore, exogenous ketone supplements that provide BHB directly without requiring strict carbohydrate restriction are being investigated as potential adjuncts or alternatives to the KD [94]. However, their efficacy and long-term safety require further validation [92,93].

Recent advancements have suggested that the effectiveness of the cKD and its application in DRE management may be significantly influenced by genetic and microbial factors. A genome-wide association study identified potential genetic markers that could predict responsiveness to the cKD, suggesting that variations in ion channels and neurotransmitter-related genes may play a crucial role [95]. Furthermore, transcriptomic analyses demonstrated that cKD alters gene expression related to epileptogenesis, thereby affecting pathways such as synaptic plasticity, neuroinflammation, and oxidative stress [91,96]. Emerging research has highlighted the interaction between the gut microbiome and the response to the cKD, thereby indicating that microbiota composition may influence ketone metabolism and neuroprotective effects [96]. These findings emphasize the need to integrate precision medicine approaches with multiomics analyses to optimize cKD strategies based on individual genetic and microbiome profiles. Future studies should aim to develop tailored dietary protocols that maximize efficacy while minimizing adverse effects to ensure a more personalized and effective therapeutic approach for DRE management [91].

Future research should focus on the development of personalized treatment protocols based on biomarkers that can predict responsiveness to the KD. Advances in multiomics approaches, including metabolomics, microbiome analysis, and neuroimaging, can help stratify patients based on their metabolic and genetic profiles, and thereby enable more targeted dietary interventions [95]. Moreover, long-term clinical trials with standardized outcome measures are required to assess the safety, adherence, and efficacy of the KD in different patient populations [91]. Although the KD and BHB represent promising therapeutic strategies for refractory epilepsy and other neurological conditions, their application must be refined through precision medicine approaches. Identifying predictive biomarkers, optimizing dietary protocols, and developing novel metabolic interventions are critical for maximizing efficacy and minimizing risks in clinical practice. Collaborative research efforts that integrate clinical, genetic, and metabolic data are crucial to advancing the KD as a mainstream therapeutic option for neurological disorders [92,93].

## 6. Conclusions

The KD and its applications have emerged as crucial therapeutic approaches for managing DRE, particularly in patients with strong genetic bases. By shifting cerebral metabolism from glucose to ketones, the KD provides an alternative energy source that bypasses the metabolic defects seen in conditions such as PDCD and GLUT1DS. Furthermore, the neuroprotective effects of the KD, including enhanced mitochondrial function, modulation of neurotransmitter balance, and anti-inflammatory properties, make it a promising intervention for a range of genetic epilepsies, including those associated with *SCN1A*, *TSC*, *CDKL5*, and *STXBP1* mutations. Although the efficacy of the KD has been well documented, its safety profile varies across patient populations and necessitates individualized treatment plans. Despite its benefits, limitations, such as inter-individual variability in responses, metabolic side effects, and difficulties in adherence, remain significant challenges. Advances in multiomics approaches, including metabolomics and genetic profiling, may enable precise medical strategies to optimize KD protocols based on patient-specific metabolic and genetic markers. Furthermore, the KD variants such as the MAD, MCTD, and LGIT offer alternative options that improve compliance while maintaining therapeutic benefits.

Future research should focus on refining dietary interventions using biomarker-driven approaches, investigating the long-term safety of sustained ketosis, and developing novel metabolic therapies, including exogenous ketone supplementation. A deeper understanding of BHB’s role in neuronal homeostasis and epigenetic regulation may reveal additional therapeutic applications beyond epilepsy. As the KD continues to gain recognition as a viable nonpharmacological intervention, collaborative efforts integrating neurology, genetics, and nutritional science are essential to maximize its efficacy and expand its clinical utility in personalized medicine.

## Figures and Tables

**Table 1:** Genetic markers that may predict the therapeutic response to the cKD.

Category	Gene	Associated Disorder	Impact on cKD Response	Mechanistic Considerations	References
Positive Response	*SLC2A1*	Glucose Transporter Type 1 Deficiency Syndrome (GLUT1DS)	Strongly Positive	Ketones provide an alternative energy source due to defective glucose transport	[39,40]
	*PDHA1*	Pyruvate Dehydrogenase Complex Deficiency (PDCD)	Strongly Positive	cKD bypasses defective pyruvate metabolism by providing ketones as an energy source	[34,35]
	*SCN1A*	Dravet Syndrome	Positive	Ketones stabilize neuronal hyperexcitability in sodium channel dysfunction	[43,45]
	*KCNQ2/KCNQ3*	KCNQ2/3-related Developmental and Epileptic Encephalopathy (DEE)	Positive	β-hydroxybutyrate enhances M-current, stabilizing neuronal excitability	[72]
	*TSC1/TSC2*	Tuberous Sclerosis Complex (TSC)	Positive	cKD reduces mTORC1 overactivation and oxidative stress	[51]
	*CDKL5*	CDKL5 Deficiency Disorder (CDD)	Variable to Positive	cKD may stabilize neuronal excitability but response varies	[53,54]
	*STXBP1*	STXBP1-related Epilepsy	Variable to Positive	Potential benefits via synaptic stabilization and mitochondrial support	[56]
	*SCN8A*	SCN8A-related Epileptic Encephalopathy	Variable to Positive	Some cases respond well, possibly through mitochondrial modulation	[67]
	*GRIN2A*	Landau–Kleffner Syndrome (LKS)	Variable to Positive	Some reports indicate benefits, but more studies are needed	[74]
	*MT-TL1*	MELAS Syndrome	Positive with Caution	cKD may improve mitochondrial function but requires careful monitoring	[63,64]
Contraindications/Cautious Use	*PC*	Pyruvate Carboxylase Deficiency	Contraindicated	cKD exacerbates metabolic acidosis due to impaired anaplerotic metabolism	[75,76]
	*SLC22A5*	Primary Carnitine Deficiency	Contraindicated	Fat metabolism dysfunction prevents ketone utilization	[8,77]
	*mtDNA deletions*	Mitochondrial DNA Deletion Syndromes (e.g., Kearns-Sayre)	Contraindicated	cKD may exacerbate metabolic decompensation, but LGIT may be safer	[63,78,79]
	*ACADM*	Medium-chain Acyl-CoA Dehydrogenase Deficiency (MCADD)	Contraindicated	cKD leads to hypoglycemia and energy deficits	[80]
	*ACADVL*	Very Long-chain Acyl-CoA Dehydrogenase Deficiency (VLCADD)	Contraindicated	Fatty acid oxidation defects cause metabolic instability	[81]
	*HADHA/HADHB*	Long-chain 3-Hydroxyacyl-CoA Dehydrogenase Deficiency (LCHADD)	Contraindicated	Long-chain fatty acid metabolism impaired, but MCTD may be cautiously considered	[82]
	*POLG*	POLG-related Mitochondrial Disorders	Highly Cautious Use	Risk of liver toxicity, metabolic crises, and worsening mitochondrial function	[63,83]

cKD, classic ketogenic diet; SLC2A1, solute carrier family 2 member 1; GLUT1DS, glucose transporter type 1 deficiency syndrome; PDHA1, pyruvate dehydrogenase E1-alpha subunit 1; PDCD, pyruvate dehydrogenase complex deficiency; SCN1A, sodium voltage-gated channel alpha subunit 1; KCNQ2, potassium voltage-gated channel subfamily Q member 2; KCNQ3, potassium voltage-gated channel subfamily Q member 3; DEE, developmental and epileptic encephalopathy; TSC1, tuberous sclerosis complex 1; TSC2, tuberous sclerosis complex 2; TSC, tuberous sclerosis complex; CDKL5, cyclin-dependent kinase-like 5; CDD, CDKL5 deficiency disorder; STXBP1, syntaxin-binding protein 1; SCN8A, sodium voltage-gated channel alpha subunit 8; GRIN2A, glutamate ionotropic receptor NMDA type subunit 2A; LKS, Landau–Kleffner syndrome; MT-TL1, mitochondrially encoded tRNA leucine 1 (UUR); MELAS, mitochondrial encephalopathy, lactic acidosis, and stroke-like episodes; PC, pyruvate carboxylase; PCCA, propionyl-CoA carboxylase subunit alpha; PCCB, propionyl-CoA carboxylase subunit beta; SLC22A5, solute carrier family 22 member 5; ACADM, acyl-CoA dehydrogenase medium-chain; MCADD, medium-chain acyl-CoA dehydrogenase deficiency; ACADVL, acyl-CoA dehydrogenase very long-chain; VLCADD, very long-chain acyl-CoA dehydrogenase deficiency; HADHA, hydroxyacyl-CoA dehydrogenase trifunctional multienzyme complex subunit alpha; HADHB, hydroxyacyl-CoA dehydrogenase trifunctional multienzyme complex subunit beta; LCHADD, long-chain 3-hydroxyacyl-CoA dehydrogenase deficiency; MCTD, medium-chain triglyceride diet; POLG, polymerase gamma; mtDNA, mitochondrial deoxyribonucleic acid.

**Table 2:** Genetic and systemic complications associated with the classic ketogenic diet (cKD) and its applications.

Complication Category	Complication	Proposed Mechanism	Genetic Associations	Implications for cKD Use	References
Metabolic	Metabolic acidosis	Impaired buffering capacity, increased ketone production, accumulation of lactate	*PC*, *SLC22A5*	Absolute contraindication due to risk of severe acidosis	[8,75]
	Hypoglycemia	Enhanced insulin sensitivity, reduced glycogen stores	No strong genetic predisposition identified	Close glucose monitoring required, particularly in young children	[8,86]
Mitochondrial Dysfunction	Worsening of energy metabolism	Inability to utilize fatty acids, increased lactate production	mtDNA deletions, *PDHA1*	Relative contraindication; LGIT may be safer due to partial glucose allowance	[63,85]
Gastrointestinal	Constipation, nausea, vomiting	Altered gut motility due to high fat intake, gut microbiota shifts	No specific monogenic link, but gut microbiota composition may influence tolerance	Dietary fiber, hydration, and probiotics may improve symptoms	[23,26,31,32,85]
	Pancreatitis	Hyperlipidemia-induced pancreatic stress, impaired lipid metabolism	Susceptibility in LPL (Lipoprotein lipase) deficiency, though rare	Avoid in individuals with a history of pancreatitis or hypertriglyceridemia	[26,85]
Cardiovascular	Prolonged QT, cardiomyopathy	Electrolyte imbalances, selenium deficiency, altered lipid metabolism	*SLC22A5* (Primary Carnitine Deficiency) may contribute to cardiac dysfunction	Requires regular ECG and selenium/carnitine monitoring	[77,85]
Renal	Nephrolithiasis	Increased urinary calcium excretion, hypocitraturia, reduced urinary citrate	No strong genetic predisposition identified	Potassium citrate supplementation recommended for prevention	[17,23,25,85]
Liver Dysfunction	Hepatic steatosis, elevated liver enzymes	Altered lipid metabolism, increased hepatic fat accumulation	Some mitochondrial disorders (*POLG*-related hepatopathy) may predispose to liver dysfunction	Regular liver function test monitoring required	[63,85]
Hematologic	Neutropenia, platelet dysfunction	Increased ketone metabolism affecting bone marrow function	No strong genetic predisposition identified	Routine blood count monitoring advised in long-term KD use	[23,85]
Neurological	Exacerbation of seizures	Inability to metabolize ketones effectively, paradoxical seizure worsening	*POLG* mutations can lead to progressive myoclonic epilepsy	Extreme caution required, as worsening seizures may occur	[79,85]
Fatty Acid Oxidation Disorders	Energy failure, metabolic crisis	Impaired β-oxidation, dependence on glucose metabolism	*ACADM*, *ACADVL*, *HADHA*, *HADHB*, *ACADS*	Absolute contraindication for cKD; MCTD may be cautiously considered in LCHAD patients under strict metabolic monitoring	[80,82,85]
Nutritional Deficiencies	Selenium, carnitine, vitamin D deficiency	Decreased intake due to dietary restrictions, increased nutrient loss	No single-gene association, but *SLC22A5* (Carnitine Transporter Deficiency) may increase carnitine loss	Routine supplementation of selenium, carnitine, and vitamin D required	[17,85]

PC, pyruvate carboxylase; SLC22A5, solute carrier family 22 member 5; mtDNA, mitochondrial DNA; PDHA1, pyruvate dehydrogenase E1-alpha subunit 1; LGIT, low glycemic index treatment; LPL, lipoprotein lipase; ECG, electrocardiogram; CLCN5, chloride voltage-gated channel 5; POLG, DNA polymerase gamma; cKD, classic ketogenic diet; MCTD, medium-chain triglyceride diet; LCHAD, long-chain 3-hydroxyacyl-CoA dehydrogenase deficiency; ACADM, acyl-CoA dehydrogenase medium-chain; ACADVL, acyl-CoA dehydrogenase very long-chain; HADHA, hydroxyacyl-CoA dehydrogenase alpha subunit; HADHB, hydroxyacyl-CoA dehydrogenase beta subunit; ACADS, acyl-CoA dehydrogenase short-chain.

## Data Availability

The data supporting the findings of this study are available from the corresponding author upon request due to the data are part of an ongoing study.

## Abbreviations

The following abbreviations are used in this manuscript:

cKD	Classic ketogenic diet
SLC2A1	Solute carrier family 2 member 1
GLUT1DS	Glucose transporter type 1 deficiency syndrome
PDHA1	Pyruvate dehydrogenase E1-alpha subunit 1
PDCD	Pyruvate dehydrogenase complex deficiency
SCN1A	Sodium voltage-gated channel alpha subunit 1
KCNQ2	Potassium voltage-gated channel subfamily Q member 2
KCNQ3	Potassium voltage-gated channel subfamily Q member 3
DEE	Developmental and epileptic encephalopathy
TSC1	Tuberous sclerosis complex 1
TSC2	Tuberous sclerosis complex 2
TSC	Tuberous sclerosis complex
CDKL5	Cyclin-dependent kinase-like 5
CDD	CDKL5 deficiency disorder
STXBP1	Syntaxin-binding protein 1
SCN8A	Sodium voltage-gated channel alpha subunit 8
GRIN2A	Glutamate ionotropic receptor NMDA type subunit 2A
LKS	Landau–Kleffner syndrome
MT-TL1	Mitochondrially encoded tRNA leucine 1 (UUR)
MELAS	Mitochondrial encephalopathy, lactic acidosis, and stroke-like episodes
PC	Pyruvate carboxylase
PCCA	Propionyl-CoA carboxylase subunit alpha
mtDNA	Mitochondrial deoxyribonucleic acid
POLG	Polymerase gamma
MCTD	Medium-chain triglyceride diet
LCHADD	Long-chain 3-hydroxyacyl-CoA dehydrogenase deficiency
PCCB	Propionyl-CoA carboxylase subunit beta
SLC22A5	Solute carrier family 22 member 5
ACADM	Acyl-CoA dehydrogenase medium-chain
MCADD	Medium-chain acyl-CoA dehydrogenase deficiency
ACADVL	Acyl-CoA dehydrogenase very long-chain
VLCADD	Very long-chain acyl-CoA dehydrogenase deficiency
HADHA	Hydroxyacyl-CoA dehydrogenase trifunctional multienzyme complex subunit alpha
HADHB	Hydroxyacyl-CoA dehydrogenase trifunctional multienzyme complex subunit beta
LGIT	Low glycemic index treatment
LPL	Lipoprotein lipase
ECG	Electrocardiogram
CLCN5	Chloride voltage-gated channel 5
MCTD	medium-chain triglyceride diet
LCHAD	long-chain 3-hydroxyacyl-CoA dehydrogenase deficiency
